# Safety and efficacy of emergency laparoscopic common bile duct exploration in elderly patients with complicated acute cholangitis

**DOI:** 10.1007/s00464-019-06914-8

**Published:** 2019-06-17

**Authors:** Baoxing Jia, Zhe Jin, Wei Han, Yahui Liu

**Affiliations:** Department of Hepatobiliary and Pancreatic Surgery, the First Hospital of Jilin University, NO. 71 Xinmin Street, Changchun, 130021 Jilin China

**Keywords:** Emergency, Acute cholangitis, Laparoscopic common bile duct exploration

## Abstract

**Background:**

Acute cholangitis (AC) is an acute inflammation of the biliary tract caused by bacterial infection, which occurs due to biliary obstruction primarily because of bile duct stones. We aimed to study the effect of laparoscopic common bile duct exploration in the treatment of complicated AC for elderly patients.

**Method:**

Elderly patients with complicated AC admitted to our hospital from August 2014 to August 2018 were considered. According to the patients’ general conditions and the American Society of Anesthesiologists’ (ASA) grade, 98 patients were divided into three groups: ASA grade II, 38 patients; ASA grade III, 33 patients; and ASA grade IV, 27 patients; all patients underwent emergency laparoscopic common bile duct exploration within 8 h of admission. The perioperative data of these patients were analyzed.

**Results:**

There were no significant differences between the three groups in preoperative laboratory test results, except for albumin levels. Conversely, when compared in every group, there were some significant differences in changes between pre- and postoperative laboratory test results, except for albumin levels. There were no significant differences between the groups in terms of perioperative data (operation time, blood loss, peritoneal drainage time, postoperative time to flatus, and postoperative hospital stay). Although four patients had postoperative complications, there were no significant differences in the rate of complications between the groups.

**Conclusion:**

Laparoscopic common bile duct exploration is a safe, effective, and feasible method for treating complicated AC in elderly patients. It should be actively used in clinical work to rapidly relieve biliary obstruction.

Acute cholangitis (AC) is an acute inflammation of the biliary tract caused by bacterial infection, occurring due to biliary obstruction primarily because of bile duct stones. The pathophysiological process of AC involves destruction of the barrier between the capillary bile duct and the liver sinusoid, resulting in sepsis, septic shock, and multiple organ dysfunction due to bacteria entering the bloodstream. If the obstruction cannot be removed in time, it often rapidly develops into acute obstructive suppurative cholangitis, which is life threatening. Therefore, biliary obstruction removal and infection control are the primary treatment measures [1–4]. With an aging global society, the proportions of elderly patients with complicated AC and systemic concomitant diseases along with AC are gradually increasing [5, 6]. At the same time, due to the development of ultrasound puncture drainage and endoscopic techniques, these patients often choose a more conservative treatment strategy to replace classic laparoscopic common bile duct exploration (LCBDE); therefore, the disease is not treated systematically and completely [7–9]. The purpose of this study was to assess the safety and effectiveness of emergency LCBDE in elderly patients.

## Materials and methods

### Clinical data

#### Inclusion criteria and general data

Elderly patients with complicated AC who were admitted to our hospital from August 2014 to August 2018 were considered. The inclusion criteria were age > 65 years, emergency admission, satisfying the diagnostic criteria for AC specified in the Tokyo Guidelines [10], higher than normal levels of leucocytes and total bilirubin (TBIL), imaging findings suggestive of calculus, extrahepatic biliary obstruction with intrahepatic and extrahepatic bile duct dilatation larger than 10 mm, and associated diseases that could lead to increased perioperative risks. Patients with a history of open biliary tract exploration or other surgery, which could interfere with classic LCBDE, were excluded.

Altogether, 98 patients met the inclusion criteria (Table 1). Informed consent was obtained from all patients, and the study was approved by the hospital institutional review board.

**Table 1:** Basic characteristics of the 98 patients

Details	
Average age (years)	74.8
Sex (male/female)	48/50
History of LC	13
Number of patients with gallstones (n)	78
Hypertension	14
Coronary heart disease	25
Myocardial ischemia	25
Chronic obstructive pulmonary disease	8
Diabetes	11
Chronic renal dysfunction	2
Posthepatitis cirrhosis	4
Cerebral infarction	2

*LC* laparoscopic cholecystectomy

#### Treatment strategy and groups

The selected 98 patients underwent active and effective anti-inflammatory fluid replacement treatment and emergency LCBDE within 8 h of admission. Thirteen patients with a history of LC underwent only LCBDE and the other patients underwent LC combined with LCBDE. The standard four-trocar technique was used, and the 12-mm main operating port was used through the epigastrium. After the common bile duct (CBD) and Calot’s triangle were clearly exposed, a longitudinal incision was made in the CBD before exploration for all patients. Normal CBD exploration was a two-step process. First, we used saline to flush the CBD and removed the stones. Second, the Olympus fiber choledochoscope was used to examine the CBD and the basket was used to remove the remnant stones. If the stones were large or fixed, we used electrohydraulic lithotripsy to break the stones before taking it out. Finally, all patients underwent choledochoscopy to confirm that there were no residual stones in the CBD. The 18-Fr or 20-Fr T-tube was used in all patients according to the inner diameter of the CBD, and a 4-0 absorbable suture was used to close the CBD incision and secure the T-tube. Saline was flushed through the T-tube to rule out leakage. A drainage tube was placed beneath the gallbladder bed and near the T-tube.

Patients were divided into three groups based on the preoperative general status and the American Society of Anesthesiologists (ASA) grade: ASA grade II with low risk (38 patients), ASA grade III with medium risk (33 patients), and ASA grade IV with high risk (27 patients). There were no statistically significant differences in sex, age, and underlying disease between the groups.

#### Clinical observation data

The three patient groups were analyzed based on laboratory data including white blood cell (WBC) count and albumin (ALB) and total bilirubin (TBIL) levels at admission and on the fifth day after surgery; their preoperative and postoperative trends were also assessed. LCBDE perioperative data, which included operation time, intraoperative blood loss, postoperative abdominal drainage time, postoperative oral feeding time (time to flatus), and postoperative hospital stay, were also evaluated, along with special situations in the LCBDE perioperative period, such as a biliary leak and intraperitoneal hemorrhage.

### Statistical analysis

Statistical data were analyzed using SPSS 22.0 (IBM Corp., Armonk, NY). Data were expressed as mean and standard deviation. Between-group comparisons were performed using both the *χ*^2^ test for numerical data and Student’s t test for measurement data to check for significant differences. If the data were not suitable for the *χ*^2^ test, Fisher’s exact probability test was used. *P* values were specified with the level of significance set at < 0.05.

## Results

### Laboratory data

Table 2 shows the comparative laboratory data for WBC count and ALB and TBIL levels at admission and on the fifth day after surgery in the three groups. There were no statistically significant differences among the three groups in terms of most of the evaluated parameters, except for preoperative differences in ALB levels among the three groups. A comparison between the three groups revealed that the preoperative ALB levels were lower in the ASA grade IV group than in the other two groups, which was representative of the poor nutritional status. Changes in laboratory test results from admission to the fifth day after surgery reflected the effectiveness of the surgical treatment and recovery of the patients during the perioperative period. As shown in Table 3, except for ALB level changes in the ASA grade II group, the other data significantly improved on the fifth day after surgery and the difference was statistically significant.

**Table 2:** Comparison of white blood cell count and albumin and total bilirubin levels at admission and on the fifth day after surgery

	Group 1 (38 patients)	Group 2 (33 patients)	Group 3 (27 patients)	*P* value
WBC count at admission (× 10^9^/L)	14.13 ± 2.42	14.66 ± 2.35	15.02 ± 3.04	0.51
ALB level at admission (g/L)	34.08 ± 4.09	32.95 ± 3.86	29.35 ± 5.09	< 0.05
TBIL level at admission (µmol/L)	92.39 ± 51.05	100.21 ± 83.49	99.14 ± 65.59	0.72
Postoperative WBC count (× 10^9^)	7.65 ± 2.29	8.00 ± 2.41	8.37 ± 2.37	0.61
Postoperative ALB level (g/L)	35.53 ± 4.33	35.31 ± 3.78	33.48 ± 4.34	0.25
Postoperative TBIL level (µmol/L)	19.33 ± 7.31	20.66 ± 7.44	20.88 ± 7.13	0.73

Group 1: ASA grade II; Group 2: ASA grade III; Group 3: ASA grade IV

Comparison of data of ALB levels among the three groups at admission; P < 0.05

*WBC* white blood cell, *ALB* albumin, *TBIL* total bilirubin

**Table 3:** Changes in white blood cell count and albumin and total bilirubin levels at admission and 5 days after surgery

Group	Admission	Fifth postoperative day	*P* value
Group 1 (38 patients)			
WBC count (× 10^9^/L)	14.13 ± 2.42	7.65 ± 2.29	< 0.05
ALB level (g/L)	34.08 ± 4.09	35.53 ± 4.33	0.17
TBIL level (µmol/L)	92.39 ± 51.05	19.33 ± 7.31	< 0.05
Group 2 (33 patients)			
WBC count (× 10^9^/L)	14.66 ± 2.35	8.00 ± 2.41	< 0.05
ALB level (g/L)	32.95 ± 3.86	35.31 ± 3.78	< 0.05
TBIL level (µmol/L)	100.21 ± 83.49	20.66 ± 7.44	< 0.05
Group 3 (27 patients)			
WBC count (× 10^9^/L)	15.02 ± 3.04	8.37 ± 2.37	< 0.05
ALB level (g/L)	29.35 ± 5.09	33.48 ± 4.34	< 0.05
TBIL level (µmol/L)	99.14 ± 65.59	20.88 ± 7.13	< 0.05

Group 1: ASA grade II; Group 2: ASA grade III; Group 3: ASA grade IV

*ALB* albumin, *TBIL* total bilirubin, *WBC* white blood cell

Based on the above statistical data, we inferred that LCBDE as a treatment method is effective in controlling infection, alleviating biliary tract obstruction, and improving metabolic liver functions. The treatment was equally effective in all patients despite differing degrees of severity in the general condition of the patients as assessed in the preoperative assessment.

### Data from the perioperative period

Table 4 represents data from the perioperative period in the three groups. The three groups showed no statistically significant difference in terms of the operation time, intraoperative blood loss, abdominal drainage time, time to flatus, and postoperative hospital stay, indicating that the patients’ conditions did not affect the efficacy of LCBDE despite the varying general preoperative conditions and different surgical risks.

**Table 4:** Perioperative data

	Group 1 (38 patients)	Group 2 (33 patients)	Group 3 (27 patients)	*P* value
Operation time (min)	83.93 ± 34.49	89.35 ± 37.52	87.65 ± 34.68	0.58
Intraoperative blood loss (mL)	35.36 ± 16.72	40.00 ± 22.86	41.76 ± 15.10	0.49
Abdominal drainage time (d)	5.25 ± 4.18	4.83 ± 2.42	4.94 ± 2.19	0.89
Time to flatus (d)	3.71 ± 2.26	3.52 ± 0.99	3.88 ± 1.54	0.81
Postoperative hospital stay (d)	10.43 ± 4.50	10.57 ± 2.09	10.82 ± 2.19	0.93

Group 1: ASA grade II; Group 2: ASA grade III; Group 3: ASA grade IV

### Special situations in LCBDE during the perioperative period

Four patients had postsurgical complications among the three groups (Table 5): two patients in the ASA grade II group, one in the ASA grade III group, and one in the ASA grade IV group. Fisher’s exact probability test showed that the incidence of complications was not statistically different between the groups. Two patients in the ASA grade II group and one patient in the ASA grade III group had mild biliary leak postoperatively (Clavien–Dindo Classification Grade II). The bile drainage volume gradually decreased after adequate drainage and proper flushing, and the mild biliary leak resolved about 10 days after surgery. The drainage tube was successfully removed when the patients had no fluid in the abdominal cavity, which was confirmed by ultrasound. One patient from the ASA grade IV group continued to bleed from the abdominal drainage tube after surgery, and routine blood routine examination showed a progressive decline in his hemoglobin level. The condition did not improve after blood transfusion. This elderly patient, who had a history of hypertension for many years, underwent laparoscopic exploratory surgery 1 day postoperatively, during which an active hemorrhage of a small blood vessel in the gallbladder bed was noted. The vessel was clamped with an absorbable hemostatic clip, and the patient’s condition subsequently improved.

**Table 5:** Complications in the perioperative period after laparoscopic common bile duct exploration

	Group 1 (38 cases)	Group 2 (33 cases)	Group 3 (27 cases)
Mild biliary leak (< 100 mL/24 h)	2	1	0
Intraperitoneal hemorrhage	0	0	1

Group 1: ASA grade II; Group 2: ASA grade III; Group 3: ASA grade IV

### Prognosis and follow-up

All the patients recovered successfully, and no biliary stones were detected by cholangiography before discharge from the hospital. After 4–6 weeks, with the T-tube clamped, the patients returned to the hospital for removal of the T-tube. None of the patients had residual stones after cholangiography and/or choledochoscopy; hence, the T-tubes were successfully removed.

All patients were followed up for varying time periods, ranging from 3 to 30 months, and they recovered well and were satisfied with the treatment, except for four patients who had a recurrence of CBD stones during this period. The average recurrence time of CBD stones in these four patients was about 1 year after LCBDE. Two patients had no clinical symptoms and the bile duct stones were found in routine physical examination. The other two patients showed symptoms of upper abdominal pain and vomiting before the stones were detected. All patients underwent MRCP, which suggested a single small stone at the end of the CBD, and recovered after endoscopic retrograde cholangiopancreatography (ERCP) stone extraction.

## Discussion

The operation for AC in the elderly should be simple, quick, and effective because the basic health status of elderly patients is usually poor. Operation methods have improved from the traditional open bile duct exploration to laparoscopic bile duct exploration and a transcystic procedure or T-tube drainage, and now ultrasound-guided puncture and endoscopic techniques have become widely accepted [11–13]. Elderly patients differ significantly in terms of their clinical symptoms, physical signs, and actual physical condition owing to their declining physiological condition and reduced immunity. Elderly patients with AC seldom exhibit the typical Charcot’s triad, especially Reynolds signs. They also have degenerated abdominal muscles and poor sensitivity, and thus, their abdominal pain is not obvious, and the clinical symptoms and signs are inconsistent with the actual condition. They may only present with blunt or distending pain, with the inflammatory response not being apparent and the increase in body temperature and WBC levels not being significant. Most elderly patients have underlying diseases such as cardiovascular and cerebrovascular diseases, which cause harmful effects on perioperative recovery. Most of the 98 elderly patients with AC enrolled in the present study had different degrees of comorbidities, which was reflected in the varied preoperative ASA scores. Some critical patients were also classified as having ASA grade IV, which is expected to be associated with poor recovery during the perioperative period. However, although there was no preoperative drainage or ERCP, emergency LCBDE achieved remarkable outcome in the present study. In the mid-1990s, Traverso [14], who had done some early LCBDE studies, recommended one-step LCBDE to treat CBD stones, even though he supported the transcystic approach. Similar to Traverso and other scholars’ view [4, 6, 15], we consider that ERCP may lead to disruption of sphincter Of Oddi and induce several postoperative complications such as pancreatitis, bleeding, and perforation.

With an increasing ASA score, the risk for surgery and anesthesia also increases. However, the perioperative data in the high-risk ASA grade IV patients in this study were not significantly worse than those in the other two groups. Firstly, there was no significant difference in operative times and intraoperative blood loss between the three groups, and the complicated condition did not increase the difficulty of the operation. Secondly, there was no significant difference between the groups in the peritoneal drainage time, postoperative time to flatus, and postoperative hospitalization days. In addition, the efficacy of LCBDE was evaluated by analyzing changes in patients’ test results. The WBC count and ALB and TBIL levels were significantly improved regardless of the patients’ preoperative criticality and ASA score, and changes in most evaluated parameters were statistically significant. The study results of Liu [5] and Zheng [6], which compared between older than 70 patients and younger than 70 patients, also mentioned that LCBDE was effective for elderly people with CBD stones. Moreover, Zhu [15] reported that LCBDE with T-tube could improve liver function effectively and rapidly. Therefore, we believe that LCBDE is a safe and effective treatment for elderly patients with complicated AC.

LCBDE has been used worldwide for nearly 20 years, and its characteristics of a small wound and quick response have been highly valued by surgeons for a long time [16, 17]. Similarly, Chinese surgeons are rapidly improving their minimally invasive treatment techniques [18], especially their stone removing techniques, during LCBDE. Al-Temimi’s study [7] suggested that the intraoperative clearance rates ranged from 75 to 100% for LCBDE, similar to the results reported in this study. Although the choledochotomy approach is a more invasive procedure than the transcystic approach, the clearance rate of choledochotomy is higher than transcystic approach [19]. And choledochotomy does not have limitations related to the anatomy of the cystic duct and ductal stone [20]. Moreover, according to the results of this study, only four abdominal complications (4.08%) occurred after LCBDE, and some literature [6, 16] reported a similar rate. In some current studies [1, 5], hemorrhage and biliary leakage were the most common complications after LCBDE, which could be cured by the conservative treatment of drainage in most cases. There are also reports [5, 19, 21] of other rare abdominal-related complications, such as pancreatitis, ileus and intraperitoneal infection or abscess. During follow-up, recurrent CBD stones in our study were found in 4 patients(4.08%). This rate was consistent with the results (1–5%) reported in most literature [1, 5, 6]. Also in the follow-up time among our patients, the biliary injury and stricture which were considered to be the serious biliary complications [21] were not be found. Therefore, with the progress of laparoscopic technology and the skilled application of choledochoscopy, surgeons can complete laparoscopic cholecystectomy and bile duct exploration in a short time, without influencing the therapeutic effect due to the slightly complicated operation technique, and can also achieve a good prognosis.

Our study has some limitations. Firstly, selection bias is inherent to the retrospective design of the study and the number of cases is also low. RCTs involving a large number of cases are needed for future studies. Second, more clinical observation data should be included in the study, to increase the value of the study results. Finally, the follow-up period is relatively short. Longer follow-up periods could help us collect patients’ long-term complications data.

In conclusion, surgeons should try to abandon the mindset of utilizing puncture drainage as a low-risk option in emergency patients. Emergency LCBDE can rapidly relieve biliary obstruction and is thus a more reasonable strategy and, to some extent, is more conducive to the recovery of elderly patients.
